# sdef: an R package to synthesize lists of significant features in related experiments

**DOI:** 10.1186/1471-2105-11-270

**Published:** 2010-05-20

**Authors:** Marta Blangiardo, Alberto Cassese, Sylvia Richardson

**Affiliations:** 1 Department of Epidemiology and Biostatistics, School of Public Health, Imperial College. St. Mary's Campus, Norfolk Place London W2 1PG, UK; 2 Department of Statistics, University of Florence, V.le Morgagni 49, 50134, Florence, Italy

## Abstract

**Background:**

In microarray studies researchers are often interested in the comparison of relevant quantities between two or more similar experiments, involving different treatments, tissues, or species. Typically each experiment reports measures of significance (e.g. *p*-values) or other measures that rank its features (e.g genes). Our objective is to find a list of features that are significant in all experiments, to be further investigated. In this paper we present an R package called **sdef**, that allows the user to quantify the evidence of communality between the experiments using previously proposed statistical methods based on the ranked lists of *p*-values. **sdef **implements two approaches that address this objective: the first is a permutation test of the maximal ratio of observed to expected common features under the hypothesis of independence between the experiments. The second approach, set in a Bayesian framework, is more flexible as it takes into account the uncertainty on the number of genes differentially expressed in each experiment.

**Results:**

We used **sdef **to re-analyze publicly available data i) on Type 2 diabetes susceptibility in mice on liver and skeletal muscle (two experiments); ii) on molecular similarities between mammalian sexes (three experiments). For the first example, we found between 68 and 104 genes commonly perturbed between the two tissues, using the two methods described above, and enrichment of the inflammation pathways, which are related to obesity and diabetes. For the second example, looking at three lists of features, we found 110 genes commonly perturbed between the three tissues, using the same two methods, and enrichment on genes involved in cell development.

**Conclusions:**

**sdef **is an R package that provides researchers with an easy and powerful methodology to find lists of features commonly perturbed in two or more experiments to be further investigated. The package is provided with plots and tables to help the user visualize and interpret the results. The Windows, Linux and MacOS versions of the package, together with the documentation are available on the website http://cran.r-project.org/web/packages/sdef/index.html.

## Background

In microarray experiments, a commonly encountered problem is the comparison of two or more similar experiments that involve different tissue/treatment/species, with the aim of finding a list of common features perturbed in all experiments. This list should highlight a restricted set of interesting features to be further investigated and validated by direct experimentation. A natural way to proceed considers the intersection of ranked lists of features from each experiment. Here the rank is based on the *p*-values associated with each experiment, but the same methodology could be applied to other measures of interest as long as they have a common scale across the experiments (e.g. correlation coefficient). Depending on the threshold chosen to declare a gene significant in each list, intersected lists of different size can be produced. The methods implemented in this package give effective ways to derive a meaningful threshold and to return one common list. To statistically assess the intersection lists, we have proposed a novel method [1], which is based on an association ratio quantifying the departure from the null hypothesis of independence between the lists. Several testing procedures were presented in [1]. The first one tests by permutations the maximal ratio between the number of significant features observed in common between the experiments and the number in common under the hypothesis of independence. The second procedure is formulated in a Bayesian framework. It uses a multinomial distribution to model the joint distribution of significant features in the set of experiments. From the output of the Bayesian analysis, several criteria for selecting the intersection list were investigated in an extensive simulation study and compared on the basis of false positives and false negatives [1].

In this paper we describe an R package, called **sdef**, that enables the user to perform the two procedures proposed, returns a table with the list of genes in common and some illustrative plots.

## Implementation

For the sake of clarity, we now briefly recall the methodology on which **sdef **is based and describe the functions of the package in the setup of two related experiments, presented in the section "Illustrative analysis: Type 2 diabetes susceptibility in mice". However, we stress that the package deals with any number of lists and we include an example about molecular similarities between mammalian sexes for three tissues (section "Illustrative example: molecular similarities between mammalian sexes") **sdef **only requires as input the *p*-values associated with the comparison performed in each experiment. In order to make the description more concrete, we phrase it in the context of differential expression (i.e. when the biological focus is on finding genes differentially expressed between two experimental conditions, e.g. in two tissues or in two species), but we emphasize that **sdef **can be used to synthesize any lists of features of interest, for instance to compare two or more relevance networks and to build a list of significant pairwise associations that are common to the two networks.

### Frequentist Test of Maximal Association Ratio

We start by ranking the lists of *p*-values for each experiment, and by defining a fine discretization of the probability scale to obtain *H *thresholds (0 ≤ *h *≤ 1). For each threshold *h*, we calculate the number of genes in common between the two experiments *O*_11 _(*h*) as well as the expected number of genes in common by chance as , where *O*_1+ _(*h*) (respectively *O*_+1 _(*h*)) is the number of genes differentially expressed in the first (second) experiment and *n *is the total number of genes in the experiments. The association ratio *T*(*h*) is defined as:(1)

It quantifies the strength of association between the lists in terms of the ratio of observed to expected, to avoid multiple testing issues. We focus attention on the ordinal statistic *T*(*h*_*max*_) = max_*h *_*T *(*h*) which represents the maximal deviation from the null model of independence between the two experiments. This maximum value is associated with a threshold *h*_*max *_on the probability measure and with a number *O*_11 _(*h*_*max *_) of genes in common which can be selected for further investigations and mined for relevant biological pathways.

The value of the ordinal statistic *T*(*h*_*max *_) is tested through a Monte Carlo permutation test and its significance is returned by a Monte Carlo *p*-value.

**Figure 1:**
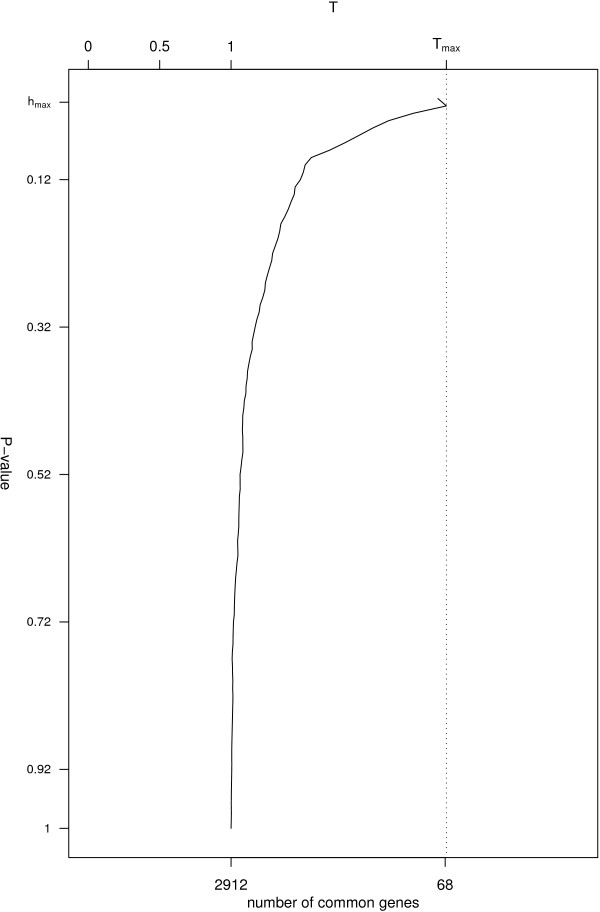
**Values of *T*(*h*) for 0 ≤ *h *≤ 1 (two lists)**. Plot for the ratio function on the mice data described in the section "Illustrative analysis: Type 2 diabetes susceptibility in mice" (two lists). The *p*-values are on the *x*-axis; the left *y*-axis shows *T*(*h*), while the right *y*-axis shows the number of genes in common for values of *T*(*h*). A dotted line is drawn for the value of *T*(*h*_*max *_), equal to 2.51, corresponding to *h*_*max *_= 0.02. In other words for a threshold of being significant of *h*_*max *_, there are 68 features with a *p*-value ≤ 0.02 that are in common between the two experiments.

The function ratio is used to obtain the statistic *T*(*h*). The data input required is in the format of a matrix where the rows are the genes, the columns are the experiments, and the cells contain *p*-values (or any suitably chosen measure to rank the features of the experiments). So, if one wishes to synthesize two experiments, on each row the first *p*-value corresponds to the significance of the statistical comparison performed in the first experiment and the second *p*-value returns the statistical significance of this comparison performed on the second experiment. The data input does not require the *p*-value to be ranked. The typical data format is presented in Table 1 and Table 2 for the examples on two and three lists. Parameters can be included to specify the directory to save the results, the name of the file and the interval of discretization. They are provided with default values. For each threshold (0 ≤ *h *≤ 1), the function ranks the features and returns the list of common genes, the number of genes differentially expressed for each experiment and the ratio *T*(*h*). Figure 1 shows the typical plot returned by the function, where *T*(*h*) is a function of the threshold *h *and a dotted line highlights the value of *T*(*h*_*max *_). The function Tmc uses Monte Carlo permutations to test if *T*(*h*_*max *_) is compatible with the null hypothesis of independence between the experiments. While the *p*-values for the first list are fixed, those for the other experiment are independently permuted *B *times. In this way, any relationship between the lists is destroyed. At each permutation *b *(1 ≤ *b *≤ *B*), *Tb*(*h*) is calculated for each *h *and a maximum statistic *Tb*(*h*_*max *_) is returned that corresponds to a sample from the null distribution of *T *(*h*_*max *_) under the condition of independence between the experiments. The relative frequency of *Tb*(*h*_*max *_) larger than *T*(*h*_*max *_) indicates where the observed *T *(*h*_*max *_) is located under the null distribution and quantifies the empirical Monte Carlo *p*-value. The user can decide the cut-off on the empirical *p*-value scale to use (usually 0.05 or 0.01 is used).

**Table 1:** Data format for sdef: two lists.

Gene	List.Pval1	List.Pval2
100005_*at*	0.936421204	0.91858576

100007_*at*	0.876117486	0.95866826

100011_*at*	0.410755946	0.06171335

100016_*at*	0.166471395	0.76881385

100024_*at*	0.008681877	0.11661176

...	...	...

The table presents the typical data format required by sdef using the mice data described in section "Illustrative analysis: Type 2 diabetes susceptibility in mice" (two lists).

**Table 2:** Data format for sdef: three lists. The table presents the typical data format required by sdef using the mice data described in the section "Illustrative analysis: molecular similarities between mammalian sexes" (three lists).

Gene	List.Pval1	List.Pval2	List.Pval3
1415670_*at*	0.01310184	0.78514374	0.3635318

1415671_*at*	0.15744532	0.40366007	0.9661227

1415672_*at*	0.01613549	0.96078200	0.1406895

141567_*at*	0.45965033	0.35167466	0.6622451

1415674_*a_at*	0.97597216	0.90075596	0.7839352

1415675_*at*	0.15111598	0.06903487	0.1528421

...	...	...	...

**Figure 2:**
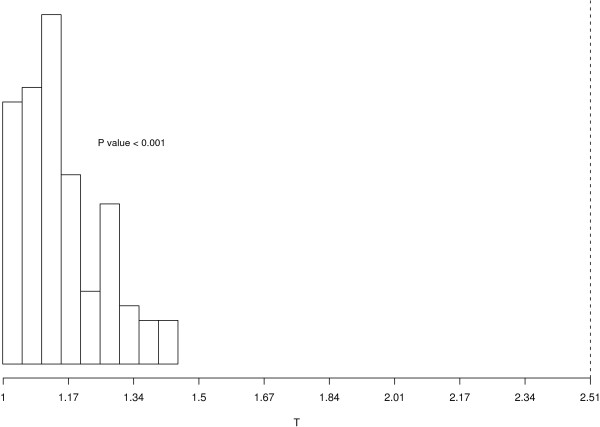
***Tb*(*h*_*max *_) distribution under the hypothesis of independence between the lists (two lists)**. Plot of the *Tb*(*h*_*max *_) distribution obtained from the Monte Carlo permutations on the mice data described in the section "Illustrative analysis: Type 2 diabetes susceptibility in mice" (two lists). The dotted line corresponds to the value of *T*(*h*_*max *_). In this case *T *(*h*_*max *_) is clearly significant as none of the statistics *Tb*(*h*_*max *_) are larger than the observed one.

The only input required for Tmc is the output from the ratio function, while the number of iterations for the Monte Carlo test is set to 1000 by default, but can be modified by the user. The function returns a histogram, presented in Figure 2, illustrating the distribution of *Tb*(*h*_*max *_) for the example on two lists. A dotted line indicates where the observed *T*(*h*_*max *_) is located with respect to the null distribution obtained through permutation.

### Bayesian Model for Association Ratio

In the second step of the analysis, we use a multinomial scenario, treating also *O*_1 _+(*h*) and *O*_+1 _(*h*) as random quantities. We specify a Multinomial-Dirichlet Bayesian model for *O*_11 _(*h*), *O*_1 _+(*h*) and *O*_+1 _(*h*). The quantity of interest is the ratio of the probability that a differentially expressed gene is truly common to both experiments, to the probability that a gene is included in the common list by chance:(2)

As the model is conjugate, it is easy to sample from the posterior distribution of *R*(*h*) given the data and to compute *CI*(*h*), the two sided Credibility Intervals for each *R*(*h*) as well as the median of the posterior distribution, *Median*(*R*(*h*)) for the desired level.

With the aim of obtaining a common list we propose to use the posterior distribution of *R*(*h*) to derive two thresholds, *h*_*max *_and *h*_2 _, which characterize respectively two decision rules. The first rule searches for the strongest deviation from independence and it is very specific (few false positives). It is obtained as the maximum of *Median*(*R*(*h*)), called *R*(*h*_*max *_) over the subset of credibility intervals which do not include the value 1 and it is equivalent to *T*(*h*_*max *_) in the frequentist framework. The second rule uses the largest threshold *h *where the number of genes called in common at least doubles the number of genes expected in common under independence (*Median*(*R*(*h*)) ≥ 2 = *R*(*h*_2 _)). It leads to a fair balance between specificity and sensitivity. See [1] for the details about the simulation studies set up to evaluate the errors associated with the two decision rules.

**Figure 3:**
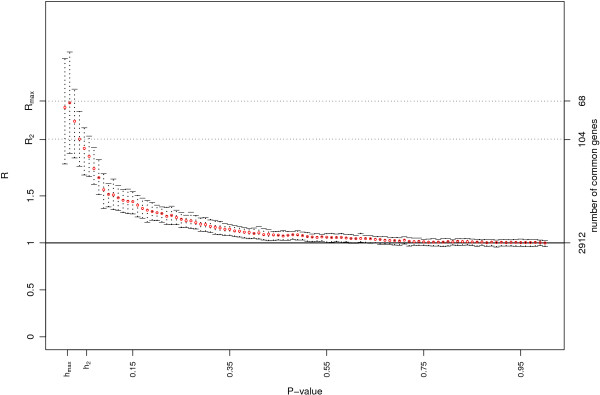
**Posterior mean of *R*(*h*) and 95% credibility interval (two lists)**. Plot for the Bayesian estimate of *R*(*h*) and its credibility interval (baymod function) on the mice data described in the section "Illustrative analysis: Type 2 diabetes susceptibility in mice" (two lists). The *p*-values are on the *x*-axis; the left *y*-axis shows *R*(*h*), while the right *y*-axis shows the number of genes in common for some values of *R*(*h*). A dotted line is drawn for the values of *R*(*h*_*max *_) and *R*(*h*_2 _). *R*(*h*_*max *_) returns a list of 68 features in common, the same as in Figure 1. *R*(*h*_2 _) corresponds to a larger list of 104 features associated with a threshold *p*-value *h*_2 _= 0.04. For this *p*-value the Bayesian model assesses that the common list of 104 features contains at least twice more genes than expected by chance.

The function baymod builds the Bayesian model described above. The input required is the output of the ratio function, and the function returns a matrix with the posterior quantiles defined by the user for *R*(*h*) (default is 2.5%, 50% and 97.5%) and a plot, presented in Figure 3 that shows the credibility intervals, and highlights the values of *R*(*h*_*max *_) and *R*(*h*_2 _) for the two decision rules. The number of iterations to estimate the posterior distribution of *R*(*h*) is 1000 by default, but can be modified by the user.

## Results

After running the Frequentist and Bayesian model, the user has to decide which model to use to obtain the list of genes in common. createTable returns a summary of the information on the degree of similarity between the experiments from the two models, and contains the rules (*h*_*max *_, *h*_2 _if available, and any additional threshold defined by the user), *T*(*h*) (only for *h*_*max *_), *R*(*h*) with its credibility interval, the number of genes in common and the number of differentially expressed genes in each experiment. Table 3 and Table 4 present the output of createTable for the data described in the Illustrative Analysis on Type 2 susceptibility in mice and for the data described in the Illustrative Analysis on molecular similarities in mammalian sexes.

**Table 3:** Common genes found using sdef: two lists.

Rule	*T*(*h*)	*R*(*h*)	***CI***_**95% **_	***O***_**11 **_	***O***_**1+ **_	***O***_**+1 **_
*h*_*max *_= 0.02	2.51	2.51	2.04 - 3.00	68	264	299

*h*_2 _= 0.04		2.11	1.81 - 2.44	104	351	410

The table shows a summary of the information on the degree of similarity between the experiments from the two models, for the mice data described in section "Illustrative analysis: Type 2 diabetes susceptibility in mice" (two lists). It is obtained running the function createTable. It contains the rules (*h*_*max*_*,h*_2 _), *T*(*h*) (only for *h*_*max *_), *R*(*h*) with its credibility interval, the number of genes in common and the number of differentially expressed genes in each experiment.

**Table 4:** Common genes found using sdef: three lists. The table shows a summary of the information on the degree of similarity between the experiments for the mice data described in the section "Illustrative analysis: molecular similarities between mammalian sexes" (three lists). It is obtained running the function createTable. It contains the rule (*h*_*max *_as *h*_2 _does not apply to this data as *R*(*h*) does not reach 2), *T*(*h*), *R*(*h*) with its credibility interval, the number of genes in common and the number of differentially expressed genes in each experiment.

Rule	*T*(*h*)	*R*(*h*)	***CI***_**95% **_	***O***_**11 **_	***O***_**1++ **_	***O***_**+1+ **_	***O***_**++1 **_
*h*_*max *_(freq & Bayesian) = 0.12	1.67	1.69	1.41 - 2.03	110	1337	2126	973

Finally, extractFeatures.T and extractFeatures.R return the list of the common genes when *h*_*max *_, *h*_2 _or an additional user defined threshold has been selected. It also creates a .csv file with the same information which can be used for further investigation, for instance to be included in softwares that perform gene enrichment (e.g. [2,3]).

### Illustrative analysis: Type 2 diabetes susceptibility in mice

We used **sdef **to re-analyze a publicly available experiment to evaluate the Type 2 diabetes susceptibility in obese and normal mice in different tissues. We focused attention on the differential expression between normal and obese mice in liver and skeletal muscle. The data are available at http://www.ncbi.nlm.nih.gov/geo, accession number GDS1443. The starting point of our methodology and the input for the R package is the matrix of *p*-values, where each row correspond to a gene (2912) and each column identifies one experiment (2 tissues). We normalized the data using the RMA function [4] implemented in the Affy R package [5] and applied Cyber-T [6] to obtain a list of *p*-values for each tissue. The format of the data matrix is presented in Table 1.

The following steps describe the use of **sdef **to find the list of common features between the two experiments. For each step we report the R code and the output. Note that this example is included in the package (Liver.Muscle function).

1. Firstly we explore the similarities between the differential expression of the two tissues through the Frequentist model. For each threshold we calculate the value of the ratio *T*(*h*)

> Th <- ratio(data)

The two outcomes for the function are:

i) a list with the number of differentially expressed genes in each experiment for each *h*, the values of the ratio *T*(*h*) and the number of genes found in common:

> Th

$h

[1] 0.01 0.02 0.03 ...

$DE

list1  list2

0.01  199  233

0.02  264  299

0.03  305  348

...

$ratios

ratio

0.01  2.449328

0.02  2.508564

0.03  2.277143

...

$common

genes in common

0.01  39

0.02  68

0.03  83

...

ii) a plot of *T*(*h*) as 0 ≤ *h *≤ 1, which is presented in Figure 1 and is saved as a .ps file in the working directory, or in the directory chosen by the user. It shows a clear association between the two lists, and it reports that there are 68 genes in common for *h*_*max *_= 0.02.

2. To compute a *p*-value for *T*(*h*_*max *_) under the hypothesis of independence between the experiments we test *T*(*h*_*max *_) using the Monte Carlo method based on permutations:

> MC <- Tmc(Th)

This is the most computationally intensive function (it takes 58 minutes to do 1000 iterations on a Dell Precision workstation with 2GB of RAM). It returns

i) an empirical *p*-value which provides the strength of the evidence that the two experiments are associated:

> MC

pvalue < 0.001

ii) a histogram which shows the distribution of *T*(*h*_*max *_) under the condition of independence between the experiments (see Figure 2). The same plot is saved as a .ps file in the working directory, or in a directory chosen by the user. From the empirical *p*-value and from the histogram it is clear in this case that *T*(*h*_*max *_) is located on the right tail of the distribution, suggesting that the data provide strong evidence of association between the two tissues in terms of differential expression. Note that for data sets with large numbers of features, we advise to use the Bayesian procedure baymod rather than the permutation test Tmc.

3. We ran the Bayesian model, which is less computationally intensive (it takes 12 minutes to do 1000 iterations on a Dell Precision workstation with 2GB of RAM):

> Rh <- baymod(Th)

The function returns

i) a table containing the posterior estimate of *R*(*h*) and its 95% credibility interval for each *h*:

> Rh

2.5%  Median  97.5%

1.8263361  2.404265  3.038746

2.0271394  2.503913  3.088150

...

ii) the corresponding plot, presented in Figure 3, where *R*(*h*_*max *_) and *R*(*h*_2 _) are highlighted. The same plot is saved as a .ps file in the working directory, or in a directory chosen by the user. As already seen for the Frequentist model, *R*(*h*) provides evidence of a clear association between the two experiments, as the credibility interval for many thresholds *h *do not include 1. *h*_*max *_remains 0.02, but *h*_2 _is 0.04, which corresponds to highlighting a list containing 104 genes in common between the two tissues. The results of the analysis are presented in Table 3.

4. Finally the list of genes in common using *h*_2 _as threshold is obtained:

> genes.R <- extractFeatures.R$rule2

$rule2

Names   List.Pval1   List.Pval2

100064_f_at  6.123493e-03   5.005709e-03

100151_at  2.255893e-03   1.454567e-03

100436_at  2.698470e-02   1.199453e-03

...

Focusing attention on this list, *CsnK2a2*, a casein kinase 2 and *Lgals3*, a galactin, have been linked to inflammatory conditions in the literature [7,8], while *atf3 *(activating transcription factor 3) and *Btg1 *(B-cell translocation gene 1, anti-proliferative) are stress-related genes; both inflammation and stress are triggered by obesity and diabetes. Moreover, *dbp *(D site albumin promoter binding protein) has been previously related to diabetes in liver and heart [9], while *Enpp2 *(autoxin) is associated to severe type 2 diabetes and linked to obesity-associated pathologies in adipose tissues [10]. Our results indicate that the role of these genes is conserved in different tissues, suggesting a systemic response that should be further investigated. **sdef **thus gives a powerful data mining tool to suggest or confirm hypotheses that require the simultaneous consideration of several experiments.

### Illustrative analysis: molecular similarities between mammalian sexes

sdef deals with any number of lists and we provide an example on three lists, re-analyzing a publicly available experiment about molecular similarities between mammalian sexes [11], which focuses attention on several tissues (hypothalamus, kidney and liver). The data are available at http://www.ncbi.nlm.nih.gov/geo, accession number GSE1147-GSE1148.

The matrix with the *p*-values contains 3 columns: i) *p*-values of differential expression between male and female mice in kidney, *p*-values of differential expression between male and female mice in liver, *p*-values of differential expression between male and female mice in reproductive system. We normalized the data using the RMA function [4] implemented in the Affy R package [5] and applied Cyber-T [6] to obtain a list of *p*-values for each tissue. We focused attention only on the present genes obtained using the mas5call function implemented in the Affy package. The total number of genes is 6477. The format of the data matrix is presented in Table 2.

The implementation of this example does not differ from what has been presented for two lists, as automatically the package recognizes the number of lists to be used by the number of columns in the data input. For this reason we do not repeat the code illustration, but we focus attention on the results. Note that this example is available as part of the R package (Example3Lists function).

**Figure 4:**
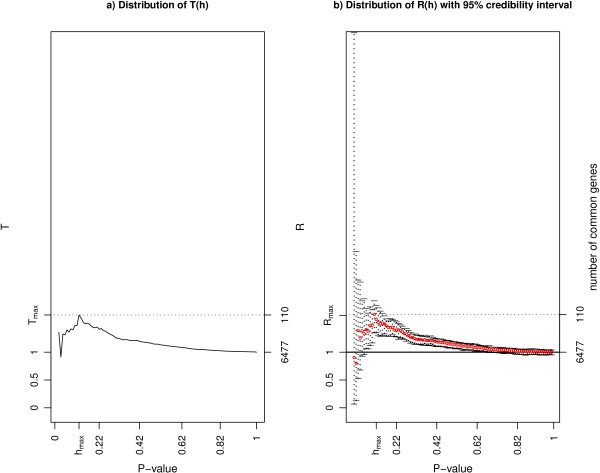
***T*(*h*) and *R*(*h*) for the illustrative example on three lists**. The figure shows a) the plot of *T*(*h*) (ratio function) and b) the plot of *R*(*h*) and its credibility interval (baymod function) on the mice data described in the section "Illustrative analysis: molecular similarities between mammalian sexes" (three lists). The *p*-values are on the *x*-axis; the left *y*-axis shows *T*(*h*) or *R*(*h*), while the right *y*-axis shows the number of genes in common for some values of *T*(*h*) or *R*(*h*). Both approaches return a list of 110 features in common for a threshold *h*_*max *_= 0.12. Note that since *R*(*h*_*max *_) < 2 there is no *R*_2 _in this example.

Table 4 and Figure 4 present the results of the analysis: 110 common genes are identified with the frequentist and Bayesian approach, with values of *T*(*h*_*max *_) = 1.67 and *R*(*h*_*max *_) = 1.69. The common genes are mostly involved in growth and cellular development (mitochondrion, nucleus) and cellular metabolic processes. Interestingly chromosome X is one of the most represented, with 5 genes which map on it (*Birc4, Btd, Gpc4, Smc1a *and *Stag2*) that are involved in sex-specific biological functions. In particular *Stag2 *and *Smc1a *are implicated in mitosis/meiosis [12] and in the maintenance of the chromosomes [13], while *Gpc4 *is responsible for the development of many organs [14], functions which are done differently for the two sexes. This suggests that some of the cellular development and maintenance mechanisms are different between the two sexes and are conserved for several tissues.

## Conclusion

**sdef **is a collection of functions to perform the comparison of two or more lists of features from similar experiments with the purpose of finding common ones to be further investigated. It is easy to use and since it needs only the lists of *p*-values as inputs it can be used to obtain results at different levels (gene level, biological function level) allowing the user to customize it to answer different types of biological questions. The methodology and the package can be applied also when a measure different from *p*-value (e.g. fold change) is used to rank the features in the experiments. However, this has an impact on the selection of the thresholds: fold changes, for instance, vary for each experiment and researchers should define a global range of values that is sensible for synthesizing all the comparisons of interest. Nevertheless the conclusions from the models would not be different using different measures of ranking, as the list of common features obtained will still contain interesting features, only based on a different measure (e.g. fold-change).

In this paper the frequentist and Bayesian approach are treated as two subsequent steps of the analysis, but we want to stress that they can be used independently from one another. The frequentist approach is an easy way to investigate the trend of *T*(*h*) and to identify how many features are found in common for different thresholds, but assessing the significance of *T*(*h*_*max *_) is extremely time consuming. Moreover, it only considers one rule (*h*_*max *_), which is more conservative and has been shown to be more affected by false negatives. The main advantage of the Bayesian approach is that it returns more accurate results through *h*_2 _and is characterized by larger lists of common features, that include all the common genes found using the frequentist approach. *h*_2 _is less affected by false negatives, but in [1] we showed that also the number of false positives remain relatively small. In addition, the Bayesian approach is extremely flexible, allowing the user to define custom thresholds, different from *h*_*max *_and *h*_2 _.

Since our methodology identifies features perturbed in two or more experiments, the proportion of false positives tends to be very small (it was around 0.5%-1.5% in the simulation presented in [1]) and the proportion is reduced as the number of lists increases. To explicitly control for false positives on the experiments under study, the user could get an estimate of the false discovery rate for each features (for instance using the method proposed by Storey in [15]) and use that as ranking statistic.

At present the package does not extend to investigate more complex patterns of association between two or more lists, for example by considering features which are perturbed only in a subset of the experiments and not in the others. This would require a modification of the methodology described in [1], which is currently under way and we plan to extend the package in the future to answer a variety of composite questions.

## Availability and requirements

**Project name **: Synthesizing Differential Expressed Genes (sdef package)

**Project home page **: http://cran.r-project.org/web/packages/sdef/index.html

**Operating systems **: Windows, Linux, MacOS

**Programming language **: R

**Other requirements **: None

**License **: GNU2

**Any restrictions to use by non-academics **: None

## Authors' contributions

MB has drafted the paper and helped with the creation of **sdef**. AC is the creator and maintainer of **sdef**, SR critically reviewed the manuscript. All authors read and approved the final manuscript.
